# Neck dissection in squamous cell carcinoma of the tongue

**DOI:** 10.1016/S1808-8694(15)31281-7

**Published:** 2015-10-20

**Authors:** Ali Amar, Otávio Alberto Curioni, Sergio Altino Franzi, Daniel Knabben Ortelado, Abrão Rapoport

**Affiliations:** 1 Department of Head and Neck Surgery and Otorhinolaryngology, Hospital Heliópolis, Hosphel, Sao Paulo, Brazil

**Keywords:** metastasis, lymph node, tonsil

## Abstract

**Aim:**

The purpose of this study was to assess the prognosis of patients with tonsillar squamous cell carcinoma with different stages of lymph node involvement and to determine the best elective neck dissection for those cases.

**Study design:**

Case series.

**Material and Method:**

51 patients with tonsillar tumors were treated between 1992 and 2001. The incidence of different tumor-node-metastasis stages was evaluated according to primary tumor extension.

**Results:**

cN0 patients had metastases in stages I and II only. Among pN+ subjects with stage I metastases, 6/7 had primary tumor extending to oral cavity.

**Conclusion:**

Supraomohyoid neck dissection (stages I, II and III) is the elective treatment of choice when tonsillar primary tumor extends to oral cavity. When primary tumors are limited to the oropharynx, selective neck dissection of stages II and III proved to be more adequate.

## INTRODUCTION

The proposed elective extended neck dissection in surgical approaches of oropharyngeal tumors remains unclear. Among selected standard neck dissections, the supraomohyoid dissection (stages I, II and III) or jugular dissection (stages II, III and IV) consist of the treatments of choice^1^. Generally, oropharyngeal tumors present stage II metastases and, less frequently, they produce stage III metastases, while incidence of lymph node involvement in other stages is low^2^. The present study aimed at assessing lymph node stages involved in tonsillar squamous cell carcinomas, taking into account primary tumor extension, in an attempt to best determine the most appropriate elective neck dissection.

## MATERIAL AND METHOD

Medical records of 51 patients with tonsillar squamous cell carcinoma treated at the Head and Neck Surgery Service of Hospital Heliópolis in the period of January 1992 and December 2001 were revised. Ages ranged from 37 to 83 years, mean age of 55. Regarding gender, there were 45 men and 6 women. All patients were initially treated by surgery, of which 49 cases included neck dissection. Traditional radical neck or radical modified dissections were performed in 40 patients, out of which 2 were bilaterally dissected; supraomohyoid dissection was performed in 7 subjects and comprehensive supraomohyoid dissection (stages I to IV) was performed in 2 patients. One subject was treated with lymphonodectomy and 1 was not treated in the neck. Concerning disease staging, patients were classified as T1 (3), T2 (20), T3 (24) and T4 (4). Postoperative radiotherapy with mean dosage of 61Gy (45 to 71 Gy) was applied in 44 patients, out of which 5 had previously been irradiated. Incidence of metastases was assessed based on different lymph node staging, according to American Head and Neck Society classification^1^. Additionally, primary tumor with extension to adjacent anatomical subsites and its correlation with lymph node involvement were assessed. Absolute numerical outcomes were expressed without the need of developing alternative hypotheses to justify the application of statistical tests, as therapeutic paradigm was uniform.

## RESULTS

Seventeen patients were staged cN0, out of which 6 were false-negatives. Among 34 cN+ patients, 28 had lymph node metastases according to histological exam. Stage II metastases were observed in 32/34 pN+ cases. Distribution of metastases according to lymph node stages is shown on Table 1. Stage Ib metastases were found in 7 cases, among which 6 had primary lesion extended to oral cavity (3 to retromolar region and 3 to tongue body). There were no stage Ia metastases among these patients. Out of 17 N0 subjects, 2 presented stage Ib metastases only. Regarding neoplasia extension to oral cavity, out of 51 studied patients, 38 (74%) had tonsil-limited lesions and 13 (26%) presented extraoral extension (Table 2).

**Table 1:** Distribution of metastases in different lymph node stages.

	N0pN+	N+pN+	Total
Ia	0/6	0/28	0/34
Ib	4/6	3/28	7/34
IIa	5/6	27/28	32/34
IIb	2/6	3/28	5/34
III	0/6	9/28	9/34
IV	0/6	4/28	4/34
V	0/6	4/28	4/34

**Table 2:** Anatomical subsites involved in tonsillar tumors*

Primary SiteSubsite	Tonsil	Anterior Pillar	Total
Tonsil	38	7	45
Anterior pillar	30	13	43
Posterior pillar	15	3	18
Soft palate	22	5	27
Base of tongue	18	4	22
Posterior wall	1	0	1
Retromolar	11	6	17
Tongue body	1	1	2
Mouth floor	4	5	9
Gingival region	6	1	7
Total of patients	38	13	51

* Involvement of several subsites in each patient.

## DISCUSSION

Selective dissections were performed to reduce morbidity related to radical dissection and they proved to be appropriate not only to stage N0 necks, but also to treat N+ necks of selected cases, with or without postoperative radiotherapy^3^. Reduced dissection extension was stimulated by positive outcomes in controlling regional disease and improvement of overall survival. Although cervical metastases were related to significantly low survival, poor outcomes mainly occur due to therapy failures on primary site and distantly, while isolated regional recurrences are early detected and treated at patients' follow-up^4^, ^5^.

Selective dissections of oropharyngeal lesions may consist of lateral or jugular (stages II, III and IV) approach or of supraomohyoid dissection (stages I, II and III). As metastases mainly occur at stages II and III, they are both acceptable alternatives. Supraomohyoid dissection has been advocated, as stage I is the only involved and lateral dissection incorrectly stages these patients, while stage IV metastases are not commonly found alone^6^. In our sample, most patients with stage I metastases presented tumor extension to oral cavity with involvement of tongue body or retromolar region. In patients with tumor limited to oropharynx, dissection in stages II and III yielded adequate cN0 neck staging. Although this is not a standard procedure, its indication is based on regional lymphatic drainage and frequent metastases distribution^2^, ^7^, ^8^, ^9^. Reduction of elective dissection may lead to investigation of sentinel lymph node, an established concept that involves wider technical knowledge.

Differently from mouth lesions, oropharyngeal lesions present higher incidence of stage IIb metastases, demanding elective dissection, despite the morbidity related to management of the accessory nerve, considering that stage IIb recurrences are hardly rescued.

Supraomohyoid dissection (stages I, II and III) has proved to be an appropriate elective neck treatment for cases of tonsillar tumors with extension to oral cavity. In N0 cases limited to oropharynx, neck dissection of stages II and III patients is the most appropriate indication.
